# Multigenerational exposure to increased temperature reduces metabolic rate but increases boldness in *Gambusia affinis*

**DOI:** 10.1002/ece3.8853

**Published:** 2022-04-19

**Authors:** Emma R. Moffett, David C. Fryxell, Kevin S. Simon

**Affiliations:** ^1^ 1415 School of Environment The University of Auckland Auckland New Zealand

**Keywords:** adaptation, metabolism, mosquitofish, pace‐of‐life, temperature, thermal history

## Abstract

Acute exposure to warming temperatures increases minimum energetic requirements in ectotherms. However, over and within multiple generations, increased temperatures may cause plastic and evolved changes that modify the temperature sensitivity of energy demand and alter individual behaviors. Here, we aimed to test whether populations recently exposed to geothermally elevated temperatures express an altered temperature sensitivity of metabolism and behavior. We expected that long‐term exposure to warming would moderate metabolic rate, reducing the temperature sensitivity of metabolism, with concomitant reductions in boldness and activity. We compared the temperature sensitivity of metabolic rate (acclimation at 20 vs. 30°C) and allometric slopes of routine, standard, and maximum metabolic rates, in addition to boldness and activity behaviors, across eight recently divergent populations of a widespread fish species (*Gambusia affinis*). Our data reveal that warm‐source populations express a reduced temperature sensitivity of metabolism, with relatively high metabolic rates at cool acclimation temperatures and relatively low metabolic rates at warm acclimation temperatures compared to ambient‐source populations. Allometric scaling of metabolism did not differ with thermal history. Across individuals from all populations combined, higher metabolic rates were associated with higher activity rates at 20°C and bolder behavior at 30°C. However, warm‐source populations displayed relatively bolder behavior at both acclimation temperatures compared to ambient‐source populations, despite their relatively low metabolic rates at warm acclimation temperatures. Overall, our data suggest that in response to warming, multigenerational exposure (e.g., plasticity, adaptation) may not result in trait change directed along a simple “pace‐of‐life syndrome” axis, instead causing relative decreases in metabolism and increases in boldness. Ultimately, our data suggest that multigenerational warming may produce a novel combination of physiological and behavioral traits, with consequences for animal performance in a warming world.

## Graphical abstract

Mosquitofish (*Gambusia affinis*) from warm‐source populations have relatively lower metabolic rates than mosquitofish from ambient‐source populations when measured at 30°C. Warm‐source populations displayed relatively more bold behavior at both acclimation temperatures, despite their relatively low metabolic rates at warm acclimation temperatures. Overall, our data suggest that in response to warming, multigenerational processes may not direct trait change along a simple “pace‐of‐life syndrome” axis, instead causing relative decreases in metabolism and increases in boldness.

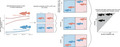

## 1 INTRODUCTION

Warming is expected to increase minimum energetic requirements and thus metabolic rate (Brown et al., 2004; Gillooly et al., 2001), potentially influencing ecologically important behaviors and the strength of top‐down effects (Angilletta & Dunham, 2003; Gardner et al., 2011; Holt & Jorgensen, 2015; Norin et al., 2016; Sibly et al., 2012). The effects of thermal change may be particularly pronounced in ectothermic species, where environmental temperature regulates body temperature. Within species, populations may respond differently to warming depending on their history of temperature exposure. For example, populations chronically exposed to elevated temperatures may exhibit altered metabolic and behavioral traits (Crozier & Hutchings, 2014). When challenged with warming environmental temperatures, these trait differences can arise quickly due to plasticity (i.e., within generation plasticity, developmental plasticity) and evolutionary adaptation, potentially mediating overall trait responses to warming (Crozier & Hutchings, 2014; Gienapp et al., 2008; Merilä & Hendry, 2014).

Population differences in the temperature dependence of metabolic rates are often studied using species distributed over altitudinal or latitudinal gradients, where adaptive change may occur over long periods (Gaitán‐Espitia & Nespolo, 2014; McKenzie et al., 2013; White et al., 2012). However, it is less clear whether adaptive change in metabolism can arise over short timescales, as could be the case under current warming. Geothermally heated habitats can offer valuable natural experiments that overcome the limitations of other natural thermal gradients and experimental approaches. For example, the use of geothermal or artificially heated waterways has recently demonstrated that long‐term exposure (e.g., 1000s of years) to increased temperatures may reduce the temperature sensitivity of metabolism in freshwater fishes (Bruneaux et al., 2014; Pilakouta et al., 2020). Similar reductions in metabolic temperature sensitivity may occur over shorter time scales (e.g., 10s to 100s of years) congruent with current environmental warming caused by climate change (Moffett et al., 2018; Sandblom et al., 2016; White & Wahl, 2020). Such moderations in metabolic rate may be associated with changes to other ecologically significant traits, such as animal behavior, but these connections are largely unknown.

Individual differences in baseline metabolic requirements may lead to consistent behavioral differences (Biro & Stamps, 2010). For example, individuals with high standard metabolic rates may also express high boldness, exploration, and activity (Bartolini et al., 2015; Biro et al., 2013; Biro & Stamps, 2010). As such, increased metabolic demand with rising temperature may be associated with an increased frequency of risk‐taking behaviors to maximize energy intake (Mathot & Dingemanse, 2015). However, selective environments may modify these plastic responses to warming. For example, if warming selects for a "fast" pace‐of‐life syndrome, individuals may evolve faster growth rates, higher metabolic rates, earlier maturation, and bolder behaviors (e.g., quicker exit of a refuge in a novel environment for foraging) (Réale et al., 2010). Alternatively, if warming selects for a "slow" pace‐of‐life syndrome (countergradient selection), then metabolism and boldness may be reduced to increase fitness through a reduction in energy expenditure, counteracting the effects of thermal plasticity alone (Réale et al., 2010; Sih et al., 2004). Individual traits may also respond in different ways to increased temperature. For example, metabolic rate may decrease while boldness increases, indicating no pace‐of‐life syndrome trait change (Morgan et al., 2020; Royauté et al., 2018). Ultimately, our ability to predict the ecological consequences of warming hinges on understanding responses of a suite of ecologically relevant traits, including metabolism and behavior.

**FIGURE 1:**
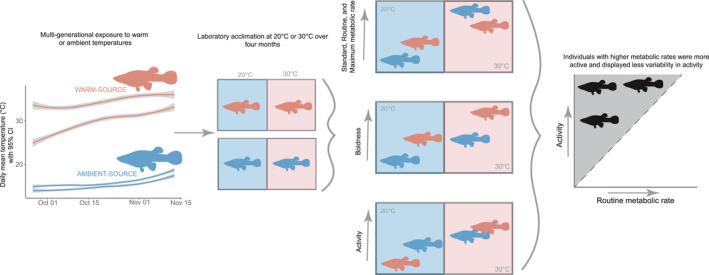
Summary of study, from left to right, *Gambusia affinis* were collected from warm‐ (pink) and ambient‐source (blue) populations and taken into the laboratory where they were acclimated at 20 or 30°C for four months. After acclimation, fish behavioral (boldness, activity) and metabolic traits (standard, routine, maximum metabolic rates) were measured and compared

Here, we use populations of a globally distributed freshwater fish, *Gambusia affinis* (hereafter *Gambusia*), to test how metabolism and behavior are affected by multiple generations of recent (~100 years) exposure to elevated temperature in natural ecosystems (Figure 1). *Gambusia* show inter‐individual and inter‐population variation in behavioral traits (Cote et al., 2010; Polverino et al., 2018) and make an ideal model organism as they have recently invaded geothermal habitats of various temperatures (Table 1) (Fryxell & Palkovacs, 2017; Moffett et al., 2018). Previous work examining *in situ* metabolic rates of *Gambusia affinis* populations across a geothermal gradient showed that the temperature sensitivity of metabolism was about seven times less than the expectation of metabolic theory (Moffett et al., 2018). This pattern suggests that (1) *Gambusia* has a low inherent temperature sensitivity of metabolism or (2) that multigenerational exposure to increased temperatures in warmer‐source populations has favored reduced metabolic rates. Here, we use laboratory acclimation of geothermal and nongeothermal populations of *Gambusia* to test the hypothesis that local adaptation (plasticity or evolution) to environmental temperature reduces metabolic rates, with a concomitant reduction in boldness and activity. This result would suggest that multigenerational exposure directs trait change in a countergradient pattern along the pace‐of‐life syndrome axis (Conover et al., 2009). Alternatively, if fish from geothermal populations show relatively low metabolism but high behavioral rates (e.g., boldness or activity), this result would suggest that multigenerational processes act to modify trait relationships, giving rise to novel trait combinations in response to warming.

**TABLE 1:** Characteristics from fish collection sites

Source	Site	Temperature at fish collection (°C)	Annual temperature range (°C)	Conductivity (µS/cm)	pH	DO (%)
Ambient	Twilight Stream	19.6	13–20	140	7.03	66
	Tahnua Torea	21.3	12–23	275	7.55	82.2
	Tourist Stream	21.8	13–23	134	6.99	51
	Auckland Domain	24.8	12–25	307	7.64	55.5
Warm	Lake Ohakuri	30.0	26–30	337	7.51	104
	Miranda Hot Spring	32.1	29–35	632	7.88	141.7
	Awakeri Spring	35.1	35–37	889	7.52	63.3
	Akatarewa Stream	36.0	24–37	483	7.43	75.5

Note

We recorded temperature, conductivity, pH, and dissolved oxygen (DO) measurements at the time of fish collection. Annual temperature range measurements were taken from bi‐monthly measurements over one year. Warm‐source fish are from geothermal systems, whereas ambient‐source populations are from systems with no geothermal influence.

## 2 MATERIALS AND METHODS

### 2.1 Fish populations and collection

*Gambusia* were introduced to New Zealand in the 1930s and spread throughout its North Island, including geothermal streams (McDowall, 1978). Assuming two generations per year (Pyke, 2008), there have been approximately 170 generations since *Gambusia* introduction to New Zealand. We collected *Gambusia* in January 2016 from eight populations in the North Island of New Zealand that differ in thermal histories (Table 1). Populations are not hydrologically connected, and we expect almost no gene flow among populations. However, due to human activity, we cannot discount some fish movement among sites. Four sites had geothermal influence and therefore had temperatures exceeding air temperature (“warm‐source”), and four sites followed changes in air temperature (“ambient‐source”). Both geothermal and ambient sites experienced daily and seasonal temperature variation. We did not have long‐term continuous temperature profiles of all sites (Figure 1 and Figure S1), but at the time of fish collection, site temperature was, on average, 11°C higher for warm‐source populations compared to ambient‐source populations. Geothermal sites reached warmer temperatures and had warmer minimum temperatures (measured bi‐monthly) than ambient sites (Table 1). Fish were collected by hand netting and transported to the laboratory in 20 L insulated buckets with water collected on‐site and a portable aerator. At the time of fish collection, we measured dissolved oxygen, pH, conductivity, and temperature using hand‐held meters (YSI Professional Plus; YSI ProODO). Fish were collected across a range of body sizes to allow for the calculation of scaling coefficients. Fish used in measurements were described as male or female based on body size, and the presence of gonopodia (male) versus a gravid spot (female).

### 2.2 Temperature acclimation

Fish from eight populations were acclimated into 16 20 L tanks (2 tanks per population) in the laboratory, with each tank containing fish from a single population. We randomly allocated ~12 fish from a population to a tank (*n* = 198 fish total; details in S1). In each tank, we separated males and females using dividers to minimize sexually antagonistic interactions that can affect survival; however, mosquitofish females store sperm, so most females were pregnant during the time of trait measurements, as they would be in nature. Each population was acclimated to two experimental temperatures (20 ± 0.5 and 30 ± 0.5°C) over four months. Tank temperatures were initially set to the collection temperature for a given population and then adjusted by increasing or decreasing the set temperature of aquarium heaters by a maximum of 1°C every two days until the target temperature was reached. We started with water from the appropriate field site in each aquarium combined with treated tap water to remove chlorine (API Stress Coat) and progressively replaced it with treated water over two weeks. We fed fish twice daily by hand to satiation with freeze‐dried *Daphnia* and Nutrafin MAX small tropical fish micro‐granules and maintained a light cycle of 12:12 throughout the experiment. Each aquarium had artificial macrophytes and stones to provide refuge. Water was continuously filtered using sponge air filters, which we cleaned every second day. Fish mortality was low in most populations (see Table S1). We fasted individuals for 24 h before measuring behavioral and metabolic traits to control for food digestion.

### 2.3 Metabolic rate

We measured metabolism as maximum metabolic rate (MMR), standard metabolic rate (SMR), and routine metabolic rate (RMR). MMR is the maximum metabolic rate of an individual and sets the upper limit on organismal metabolic performance (Fry, 1971). In contrast, SMR is the minimum metabolic rate, measured after rest, with no digestion cost, on non‐stressed fish and sets the lower requirement of an animal to sustain life. RMR was measured under similar conditions as SMR but allowed for some activity and sits between SMR and MMR. As RMR incorporates variation in activity between individuals, it may closely relate to behavioral traits (Mathot & Dingemanse, 2015).

We measured RMR and MMR using static respirometry and SMR using intermittent flow‐through respirometry at each fish's acclimation temperature (Clark et al., 2013; Steffensen, 1989). We used respirometers comprising 40 ml acrylic chambers with magnetic stir bars in the chamber base to ensure water mixing throughout our oxygen measures in all assays. We measured metabolic rate as oxygen consumption (MO_2_) using a FireSting four‐channel oxygen logger with optical oxygen sensors (PyroScience, Germany). Respirometers were placed into 80 L aquaria, filled with treated tap water, fitted with a UV filtration system, an aerator, and a 100W aquarium heater.

Immediately following behavioral trials (see below), we measured RMR by placing individuals into chambers and measuring oxygen consumption over 15 min. Chambers were then connected to a recirculating pump and slowly flushed with oxygenated water for five minutes before beginning SMR measurements. Oxygen consumption measurements for SMR were taken overnight over an approximately 18‐h period. A computer‐controlled aquarium pump intermittently flushed chambers for five minutes to ensure a complete turnover of water inside the chambers, then an oxygen measurement period of 15 min began after a 30 s wait period. We controlled oxygen flow and data logging through a PC using the software “AquaResp” (Svendsen, 2017). Following SMR measurements, we measured MMR using an exhaustive chase protocol to induce maximum oxygen consumption (Clark et al., 2013; Norin & Clark, 2016). Fish were removed from chambers one by one and placed into a circular tank; in this tank, we used an aquarium net to chase the fish until exhaustion (defined as the lack of ability for burst swimming) (Norin & Clark, 2016). Fish were then immediately placed into a static respirometer, and oxygen consumption was measured for 5 min. We chose to measure MMR after SMR measurement to ensure our SMR measurement accuracy as metabolic rates may remain elevated for long periods after exhaustive exercise. We immediately euthanized the fish following the measurement of MMR using clove oil. Fish were then measured for mass, length, sex, and volume, then dried at 60°C for 48 h and re‐weighed for dry mass.

We controlled for microbial oxygen consumption in our metabolism assay water by subtracting the oxygen consumption in blanks (respirometers with water only), which were run before and after every trial. We assumed a linear increase in microbial oxygen consumption between measurements in blanks.

We calculated each SMR, MMR, and RMR as

$${\text{MO}}_{2}=\left(V_{r}‐V_{f}\right)\times \frac{\Delta C_{\text{wO}2}}{\Delta t}$$

where MO_2_ is oxygen consumption rate, *V*_r_ is respirometer volume, *V*_f_ is fish volume, Δ*C*_wO2_ is the change in oxygen concentration, and Δ*t* is the change in time.

We calculated SMR using the mean of the lowest 10% of all measurements, excluding any outliers (± two standard deviations [SD] from the mean), aerobic scope as MMR‐SMR, and factorial aerobic scope as MMR/SMR (Chabot et al., 2016; Clark et al., 2013).

### 2.4 Behavior

Immediately before measuring metabolism, we conducted behavioral assays on individuals in a 60 L aquarium with a water depth of 20 cm and temperature set to the acclimation temperature. We fit the aquarium with an air pump and a UV filtration system to maintain high oxygen saturation and control microbial respiration. We measured individual “boldness” as latency to exit a refuge and individual “activity” as time spent exploring a novel environment (Cote et al., 2010; Wilson et al., 2010). For these behavioral measures, we placed individuals into a small enclosed and darkened area (“refuge”, 10 cm × 30 cm) at one end of the 60 L aquarium. The aquarium was covered on all but one side to allow for observation. In the refuge, we provided artificial macrophytes and river stones. Fish were left in the refuge for 10 min before a 4 × 4 cm door was opened remotely, allowing fish to exit and explore the remainder of the tank (“open area”). In the open area, we placed macrophytes opposite the refuge opening as a visual cue for exploration. We measured boldness using a stopwatch as the time it took the fish to leave the refuge. Fish that did not leave were assigned a maximum latency time of 600 s and were not measured for activity as forced tests may measure anxiety or fear traits (Brown et al., 2007). Once the fish began exploring, we video‐recorded their movement and later measured activity as time spent moving (vs. remaining stationary) over five minutes following their emergence from the refuge.

### 2.5 Statistical analysis

#### 2.5.1 Overview

We constructed models using “lme4” v.1.1.23 and calculated p values using “LmerTest”v.3.1.2 package in R with the Satterthwaite's degrees of freedom method (Bates et al., 2015; Kuznetsova et al., 2017). We performed all analyses using R version 4.0.0 and determined the results to be statistically significant at the cut‐off value *α* = 0.05 (R Development Core Team, 2020).

We first constructed a full model incorporating all predictors (mass, acclimation temperature, and thermal history as geothermal or ambient) and their interactions to analyze the influence of our predictors on metabolism and behavior. We included random effects for population identity and tank number and incorporated a null effect model for model comparisons (model 7, Table 2). We used the Akaike Information Criterion (AIC) to reduce these models (Mazerolle, 2019; Säfken et al., 2018). We ranked models by conditional Akaike Information criterion (AICc) values and averaged candidate models with ΔAICc < 4 using the R package “MuMin” v.1.43.17 and removed models with interaction terms that were not significant (Barton, 2020; Burnham & Anderson, 2001) (Tables S2 and S3). Second, for each metric of metabolism, we used linear regression on subsets of the log‐log transformed data to calculate metabolic parameters *b* and Ea (described below), as is standard practice for analyzing metabolism data (Gillooly et al., 2001; Tattersall et al., 2012). Third, we tested the relationship between metabolism and behavior across individuals from all populations combined. To do so, we related metabolic rate to boldness and activity.

**TABLE 2:** Candidate models used in model selection for factors that influence fish metabolic and behavioral traits, we included the random effects population and tank ID in each model. The seventh model is a null model

Model number	Model factors
1	Trait ~mass + thermal history + acclimation temperature
2	Trait ~mass × thermal history × acclimation temperature
3	Trait ~mass × thermal history + acclimation temperature
4	Trait ~mass + thermal history × acclimation temperature
5	Trait ~mass + thermal history + acclimation temperature + thermal history: acclimation temperature + mass: acclimation temperature
6	Trait ~mass + thermal history + acclimation temperature + mass: acclimation temperature + thermal history: mass
7	Trait ~mass + 1

#### 2.5.2 Metabolism

To understand the relationship between metabolic traits (SMR, RMR, MMR, AS) and acclimation temperature or thermal history, we used a linear mixed effect model (LMM) with mass, thermal history, and acclimation temperature included as predictor variables. We chose to exclude the factor sex in preliminary analyses because sex was not significant in determining four of the five models used for metabolic traits, and males and females have overlapping covariate ranges (Figure S3, Table S6). Further, sex is correlated with body size in this species (females are larger, Pyke, 2008).

We calculated allometric scaling coefficients (slope, *b*) using least‐squares linear regression models of log_10_ metabolic rate (µg O_2_ min^−1^) data against log_10_ mass (mg) data, separately for each thermal history ×acclimation temperature combination (*n* = 4). The activation energy (Ea) of metabolism was calculated from Arrhenius plots of mass‐normalized metabolic rates (MO_2_ × *M* ^−^ *^b^*), against acclimation temperature as an inverse function (1/*kT*), where *T* is the respirometry temperature (same as acclimation temperature) in degrees Kelvin, *k* is the Boltzmann constant (8.62 × 10^−5^ eV K^−1^), and *M* is mass as in Gillooly et al. (2001).

#### 2.5.3 Behavior

We used a mixed‐effects binomial logistic model to understand if mass, thermal history, and acclimation temperature influenced boldness. We chose this model because fish had a maximum latency time of 600 s, and, as such the upper limit of latency was unknown. Then, taking individuals that left the refuge, we used a Poisson‐lognormal generalized linear mixed‐effects model to understand if mass, thermal history, and acclimation temperature influenced activity. Like our metabolism models, we chose to exclude sex to avoid confounding trends between sex and body size (see Figure S4 for data split by sex).

To understand if boldness was associated with mass and SMR, MMR, or RMR, we used censored regression models using “censReg” v. 0.5.30 (Henningsen, 2020). Similarly, to understand if behavioral activity was related to mass and SMR, MMR, or RMR we used a Poisson‐lognormal generalized linear mixed‐effects model with source population and tank number as random effects. We constructed separate models for each acclimation temperature. We did this to avoid confounding between metabolic rate and acclimation temperature.

## 3 RESULTS

Based on model selection criteria, our best models for standard metabolic rate (SMR) (model 5), routine metabolic rate (RMR) (model 5), and maximum metabolic rate (MMR) (models 4, 1, and 5) included history × acclimation temperature and mass × acclimation temperature interactions. For AS, the best models (1, 7) included only main effects and a null model (see Table 2 for candidate models).

### 3.1 Allometric scaling of metabolism

**FIGURE 2:**
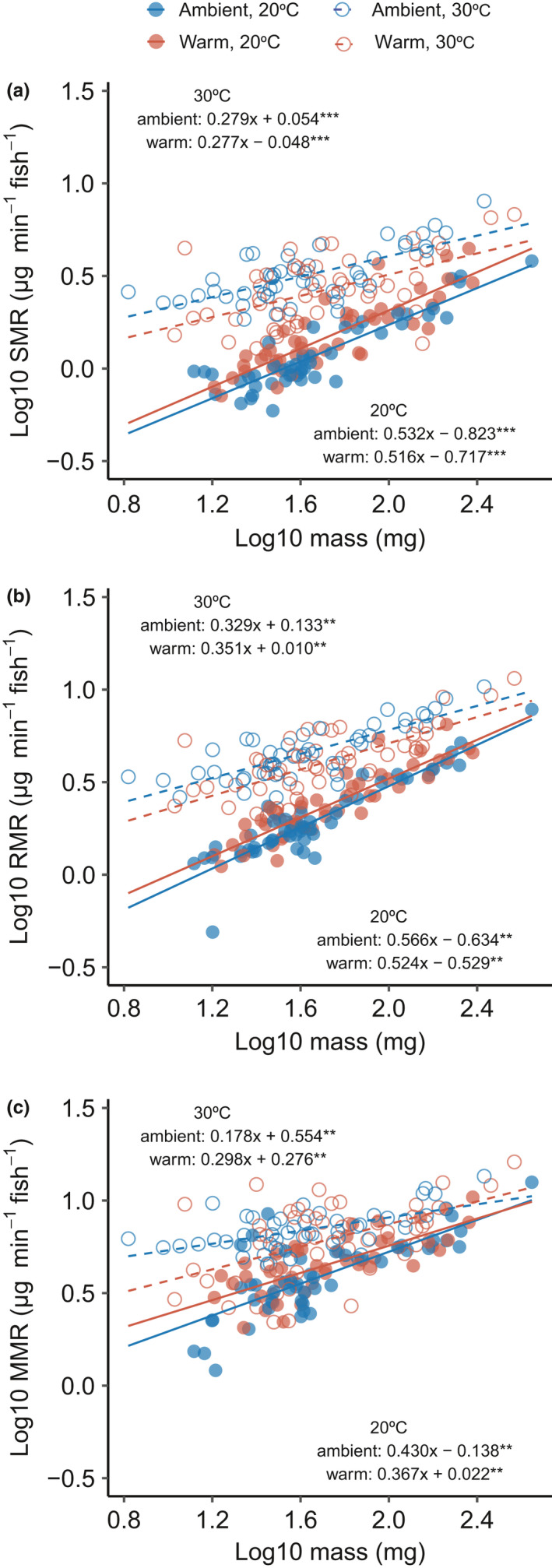
Relationship between fish mass and (a) standard metabolic rate (SMR), (b) routine metabolic rate (RMR), and (c) maximum metabolic rate (MMR) in *Gambusia affinis*. Dashed lines and open circles represent acclimation at 20°C, and solid lines and filled circles represent acclimation at 30°C. Warm and ambient refer to the population's thermal history (see Table 1). We fit data with simple linear regression models and denoted significance of these models as: <0.0001 ‘***’, <0.001 ‘**’, *n* = 198/ trait

We found a significant relationship between metabolic rate, mass, and acclimation temperature for SMR, RMR, and MMR (mass × acclimation temperature, Figure 2, Table S4). In particular, metabolism rose less with increasing mass at 30°C than at 20°C (Figure 1). The metabolic rates of smaller individuals were most sensitive to increasing acclimation temperature, as metabolic rates converged between acclimation temperatures for larger fish (Figure 1). Allometric slopes were similar between ambient‐ and warm‐source fish at each of the acclimation treatment temperatures. Across all metabolic rate measurements, scaling exponents varied from 0.178 to 0.556 and were lowest for MMR (Table S7). Aerobic scope increased with mass and was higher when fish were acclimated at 30°C compared to 20°C (Table S4). The three measures of metabolic rate were related (linear regression, *r* ^2^ = .596–0.829; Figure S2). Overall, MMR was 1.6 and 1.8 times greater than SMR for fish at 30°C and 20°C, respectively. RMR was 1.3 and 1.4 times greater than SMR for fish at 30 and 20°C, respectively (Figure S2). Across all individual fish, factorial aerobic scope values ranged from 1.4 to 9.1.

### 3.2 Temperature sensitivity of metabolism

The effect of thermal history on all metabolic rate measures (SMR, RMR, and MMR) depended on acclimation temperature (i.e., significant thermal history × acclimation temperature interactions, *p* < .001, Table S4). Individuals from warm‐source populations had lower metabolic rates than individuals from ambient‐source populations at 30°C, but the reverse was true at 20°C (Figure 1a–c). We found no relationship between aerobic scope and thermal history (*p* = .902).

**FIGURE 3:**
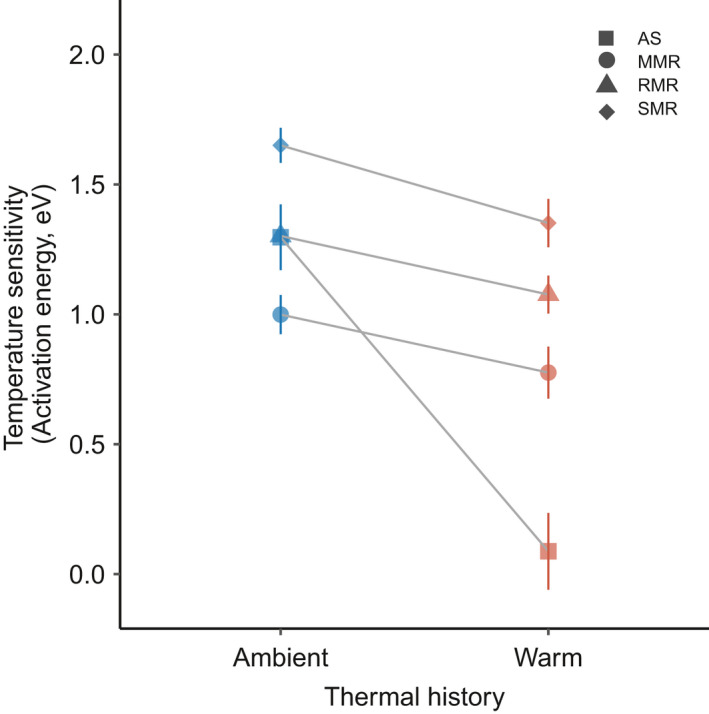
Temperature sensitivity of metabolism across laboratory acclimated ambient‐ and warm‐source populations of *Gambusia affinis*. We show temperature sensitives for each measured metabolic trait separately. Data are averages ±1 SE; *n* = 8

Similarly, temperature sensitivity (as activation energy, Ea) varied with population and acclimation temperature, where metabolic rates of individuals from warm‐source populations had lower Ea compared to individuals from ambient populations (Figure 3). Activation energies ranged from −1.655 to −0.998 eV for ambient‐source fish and from −1.351 to −0.775 eV for warm‐source fish.

### 3.3 Behavior

**FIGURE 4:**
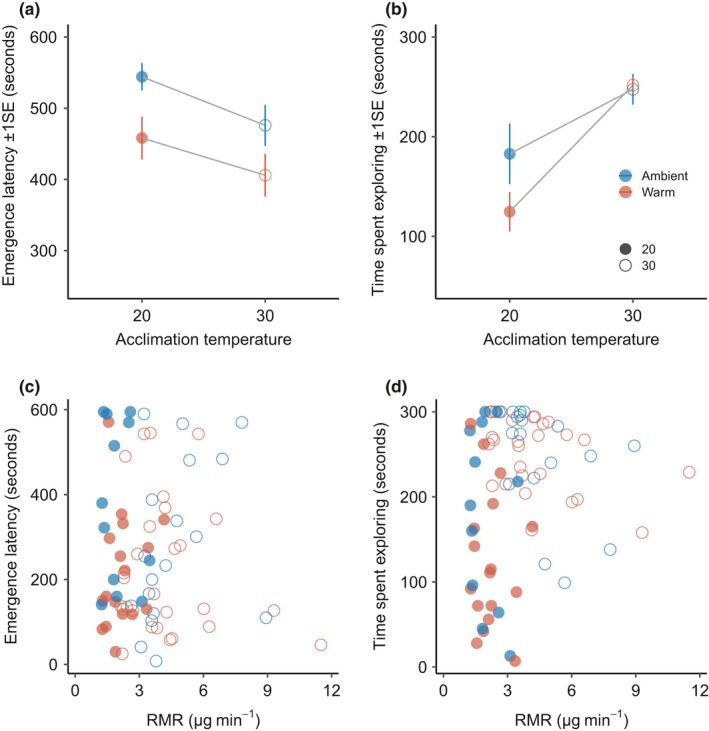
Relationship between thermal history and behavior as (a) boldness (i.e., emergence latency) and (b) activity (i.e., time spent exploring) as population differences. Plots (c) and (d) show the relationship between routine metabolic rate (RMR) and behavior as boldness and activity, respectively. We do not show individuals who did not leave the refuge, *n* = 76

Our best‐supported model for boldness was model 1, which included only main effects (Table 2). Fewer individuals from ambient‐source populations left the refuge compared to warm‐source populations (*n* = 29 and 76 respectively) (Figure 4). Individuals were bolder when they were smaller (*z* = −0.972, *p* = .044), originated from a warm‐source population (*z* = 0.718, *p* = .044), and were acclimated to 30°C (*z* = 0.061, *p* = .046) (Table S5).

The best‐supported models for activity were models 2, 5, and 4, which included interactions among all factors (Table 2). We found that the effect of thermal history on activity depended on acclimation temperature (*z* = 2.144, *p* = .032), where fish from warm‐source populations were less active when acclimated at 20°C compared to fish from ambient‐source populations. We also found an interaction among mass, thermal history, and acclimation temperature on activity (*z* = 2.321, *p* = .020). As such, we show that smaller individuals acclimated at 30°C were more active than larger individuals acclimated at 20°C and that activity was higher for cool‐source fish at 20°C than warm‐source fish at 20°C.

Boldness was not well explained by mass or metabolic rate across the two temperatures (Table S8; Figure 4). When fish were acclimated at 20°C, boldness increased with mass, but this relationship was only significant for SMR (*t* = 2.292, *p* = .022) and not for models with RMR or MMR as dependent variables (Table S8). At 30°C, fish with higher RMR were bolder (*t* = 2.244, *p* = .025), but SMR and MMR were unrelated to boldness (SMR, *t* = 1.828, *p* = .068; MMR; *t* = 1.683, *p* = .092). In contrast to boldness, activity was explained by mass and metabolic rate and their interaction, but the pattern differed between temperatures (Table S9). When acclimated at 20°C, fish with higher RMRs or MMRs and larger body size were more active (RMR: *t* = −3.431, *p* = .0006; MMR: *t* = −4.007, *p* < .0001). At 20°C, activity was not related to SMR but decreased with increasing fish mass (*t* = −4.130, *p* < .0001). At 30°C, no measures of metabolic rate were related to activity, and activity decreased with fish mass in two models (SMR: *t* = −3.208, *p* = .001; MMR: *t* = −0.12, *p* = .003) and was not significant in the other (RMR: *t* = −1.237, *p* = .216). Boldness was infrequently related to metabolic rate. When fish were acclimated at 30°C, those with higher RMRs (*t* = 2.244, *p* = .025) were bolder, but this pattern was non‐significant for other measures of metabolic rate (SMR, *t* = 1.828, *p* = .068; MMR; *t* = 1.683, *p* = .092), and at 20°C there was no relationship between boldness and any measure of metabolic rate.

## 4 DISCUSSION

Multigenerational exposure to increased temperatures may alter the temperature dependence of physiological and behavioral traits; however, the temperature dependence of physiological traits is not often examined after multiple generations of exposure to changed temperatures (Cheung et al., 2012; Holt & Jorgensen, 2015; Persson et al., 1998; West et al., 1997). Here, our results demonstrate that populations with a recent and multigenerational history of exposure to warmer temperatures (i.e., geothermal source populations) display a significantly reduced temperature sensitivity of metabolism. Moreover, at warmer acclimation temperatures, populations with a warmer thermal history show lower metabolic rates than populations from ambient conditions, suggesting that multigenerational processes (e.g., plasticity, adaptation) may counteract the metabolic consequences of temperature rise (Jutfelt, 2020; Sandblom et al., 2016). Further, we show that boldness and activity were positively related to metabolic rates across individuals from all populations combined at the individual level. However, when comparing populations, fish from warmer source populations showed relatively high boldness at both acclimation temperatures despite relatively low metabolic rates at the warmer acclimation temperature. Together, these results suggest that multigenerational warming will cause a reduction in metabolic rate and an increase in boldness and activity, but that multigenerational processes may not act to direct these trait changes neatly along a "pace‐of‐life syndrome" axis.

### 4.1 Allometric scaling and temperature sensitivity of metabolism

In *Gambusia*, allometric slopes changed with acclimation temperature, and this change was similar between thermal histories (Figure 2). Allometric slopes were shallower at the warm acclimation temperature, with small fish showing the largest difference in metabolic rates between acclimation temperatures. This difference in allometric slopes indicates that increased temperature may influence smaller individuals to a greater extent than larger individuals. The temperature‐size rule demonstrates a reduction in body size with warming in ectotherms, which *Gambusia* show (Fryxell et al., 2020; Gardner et al., 2011; Moffett et al., 2018). As such, our data suggest that the effect of warming on *Gambusia* metabolism will be the most pronounced at smaller body sizes. This increased temperature sensitivity of metabolism of small individuals with warming may have significant consequences for population size‐structure (e.g., mortality/reproduction) and the strength of top‐down effects (e.g., consumption) as energetic demand increases parallel to declines in body size (Biro et al., 2007; Fryxell et al., 2020).

Our data suggest that multigenerational exposure to warm temperatures reduced the minimum energetic requirements of metabolism (SMR) at the warm acclimation temperature (Figure 2). Similarly, sticklebacks (*Gasterosteus aculeatus*) and European perch (*Perca fluviatilis*, L.) show a reduction in SMR with a warm thermal history, indicating that such changes may be a common consequence of elevated thermal histories (Pilakouta et al., 2020; Sandblom et al., 2016). While metabolic change in sticklebacks occurred over a long period, change in the European perch was rapid, occurring over three decades. Here, change in metabolic rate in *Gambusia* occurred rapidly over ~100 years or ~170 generations (NLNZ, 1928; Pyke, 2008). Previous work with *Gambusia* in the wild demonstrated that metabolic temperature sensitivity was about seven times less than predicted by metabolic theory (Moffett et al., 2018). Here, our data suggest this discrepancy may be explained by multigenerational processes acting to reduce the metabolic rate of warm‐exposed populations over multiple generations. Similarly, the metabolic rates of coral reef fishes originating from high latitudes were less sensitive to warming than those from low latitudes, suggesting such patterns may be widespread (Munday et al., 2012).

Further, we found that metabolic rates were relatively low for warm‐source individuals when measured at 30°C but were relatively high at 20°C, demonstrating that thermal history can modify plastic responses to temperature itself. In contrast, metabolic rates in warm‐source Stickleback were consistently lower than cool‐source regardless of assay temperature (Pilakouta et al., 2020). In this study, the differences in metabolic rates with acclimation temperature may indicate a trade‐off associated with warm adaptation. For example, if warm‐source fish evolved downregulation of enzymatic and mitochondrial density to save energy at warmer temperatures, this downregulation in mitochondrial density may lead to a lower ability to cold acclimate and to function well at cooler temperatures (Salin et al., 2015).

The moderating effects of thermal history on the temperature sensitivity of metabolism were consistent across all three metabolic rate measurements. However, measurements of individual maximum metabolic rate showed the least variation between acclimation temperatures. Accordingly, our data suggest that minimum energy requirements may be more plastic than maximum energy requirements. Thermal tolerance may often evolve asymmetrically, for example, a species may show greater variation in their ability to adapt to cold temperatures than warm temperatures via greater thermal compensation in SMR compared to MMR (Addo‐Bediako et al., 2000; Araújo et al., 2013; Bennett et al., 2021; Sandblom et al., 2016). Here, factorial aerobic scope (MMR/SMR) values in our study were somewhat low, ranging from 1.4 to 9.1, whereas in teleost fishes, values ranged from 1.8 to 12.4 (Killen et al., 2016). Lower factorial aerobic scope values may suggest that the exhaustive chase protocol used to measure MMR underestimated actual MMR (Andersson et al., 2020). Nevertheless, any asymmetry in metabolic measures (SMR, RMR, MMR) could limit performance under warming if adaptive change is slow (Sterner & George, 2000).

### 4.2 Behavior, metabolism, and temperature

Fish with higher metabolic rates were more active, but this result was only apparent when fish were acclimated at 20°C. Studies that have assessed the link between metabolism and behavior have found mixed support for a relationship between these factors (Biro & Stamps, 2010; Niemela & Dingemanse, 2018; Royauté et al., 2018). Here, our data indicate that rising metabolic rate may play a significant role in regulating behavior, where, as metabolic rate increases, activity time also increases. For example, individuals with higher metabolic rates were more active and displayed less variability in activity at 20°C, with no relationship apparent at 30°C (Figure 4). Across all metabolic rate measurements, individual behavior was most consistently related to RMR, suggesting that physiological measures may be best related to behavior when they allow for variation in behavior rather than control (Careau et al., 2008). For example, RMR was the only metabolic measurement that predicted boldness, suggesting that RMR better captures behavioral variation and that risk‐taking behaviors might become more common with warmer temperatures. Here, RMR measurement likely included some recovery from handling stress and any effect of being in a new environment. As such, the significant relationship between RMR and behavior may have resulted from muscle oxygen consumption, as activity was likely a significant component of RMR measurements. Future studies would benefit from incorporating repeated measurements of behavior on each individual to disentangle the role of within‐individual variation in behavior to metabolic rate. Overall, our data suggest that increasing metabolic demand increases risk‐taking and minimal behavioral activity as temperatures warm.

Despite the positive relationships among acclimation temperature, metabolic rate, and behavior across all fish combined, we found these relationships to differ based on thermal history. If multigenerational processes had caused trait change along a pace‐of‐life syndrome axis, populations with relatively low metabolic rates should have shown low boldness and activity (Réale et al., 2010). However, warm‐source populations displayed relatively bold behavior at both acclimation temperatures, despite their relatively low metabolic rates at warm acclimation temperatures (Figure 4). Patterns in activity were less clear, as warm‐source individuals were less active than ambient‐source individuals when measured at 20°C, while at 30°C, patterns were similar. Such patterns in activity with different thermal histories may be further evidence of a significant trade‐off with warm adaptation, which may not only affect metabolic performance at lower temperatures (see above) but also behavior. Thus, warmer environments seem to favor risk‐taking behavior while reducing routine activity, perhaps to conserve energy when resources are not perceived to be readily available. Other studies typically show an increase in boldness and activity with increased environmental or acclimation temperature (Biro et al., 2010; Careau et al., 2008; Forsatkar et al., 2016), but they rarely account for population differences. From our study, it appears that multigenerational processes may not direct trait change along a simple pace‐of‐life syndrome axis, instead producing a novel combination of physiological and behavioral traits.

Our results suggest that multiple generations of exposure to warmed temperatures result in significant changes to animal physiology and behavior. Several processes may have generated the trait changes discovered here, including developmental plasticity, transgenerational plasticity, and evolution. However, ultimately, the net effect of these processes will determine the outcomes of warming for individuals and populations. Importantly, our results show that the combination of these processes over several generations tends to counteract the increase in metabolism caused by acclimation to warm temperatures while intensifying acclimatory changes in some behavioral traits. This result calls to question the notion that warming will cause trait changes along a neat pace‐of‐life syndrome axis. Clearly, we must understand the novel trait combinations arising over multiple generations of exposure to warming to better predict outcomes for the ecology of individuals, populations, and ecosystems.

## CONFLICT OF INTEREST

We declare we have no conflict of interest.

## AUTHOR CONTRIBUTIONS

**Emma R. Moffett:** Conceptualization (equal); Data curation (equal); Formal analysis (equal); Funding acquisition (equal); Investigation (equal); Methodology (equal); Project administration (equal); Validation (equal); Visualization (equal); Writing – original draft (equal); Writing – review & editing (equal). **David C. Fryxell:** Conceptualization (equal); Formal analysis (equal); Methodology (equal); Validation (equal); Visualization (equal); Writing – review & editing (equal). **Kevin S. Simon:** Conceptualization (equal); Formal analysis (equal); Investigation (equal); Methodology (equal); Resources (equal); Supervision (equal); Validation (equal); Writing – review & editing (equal).

### OPEN RESEARCH BADGES

This article has earned an Open Data Badge for making publicly available the digitally‐shareable data necessary to reproduce the reported results. The data are available at https://doi.org/10.7280/D1MT39.

## Supporting information

Supplementary Material

## Data Availability

All relevant data are archived at Dryad Digital Repository, https://doi.org/10.7280/D1MT39.
