# Supplementary material for: Multigenerational exposure to increased temperature reduces metabolic rate but increases boldness in Gambusia affinis
Source: Ecol Evol. 2022 Apr 19;12(4):e8853. doi: 10.1002/ece3.8853 (PMC9019145; doi:10.1002/ece3.8853)
Supplement: Supplementary file 1 — Supplementary Material [file ECE3-12-e8853-s001.docx]

Electronic Supplementary material for “Multigenerational exposure to increased temperature reduces metabolic rate but increases boldness.”

**Table S1.** Summary of the number of individuals we used to measure SMR, RMR, MMR, and behaviour from each population and acclimation temperature, *n* = 198.

| Site | Acclimation Temperature (°C) | The total number of fish used. |
| --- | --- | --- |
| Twilight Stream | 20 | 11 |
|  | 30 | 12 |
| Tahnua Torea | 20 | 12 |
|  | 30 | 12 |
| Tourist Stream | 20 | 14 |
|  | 30 | 12 |
| Auckland Domain | 20 | 12 |
|  | 30 | 12 |
| Lake Ohakuri | 20 | 12 |
|  | 30 | 14 |
| Akatarewa Stream | 20 | 10 |
|  | 30 | 11 |
| Awakeri Spring | 20 | 11 |
|  | 30 | 12 |
| Miranda Hot Spring | 20 | 13 |
|  | 30 | 18 |

**Table S2.** Model selection using conditional Akaike information criterion (AICc) values for each metabolic trait, standard (SMR), routine (RMR), maximum (MMR) metabolic rates, and aerobic scope (AS). Models for each trait are ranked by cAIC values. Best-fit (ΔAICc < 4) models are bolded. Model parameters are described in Table 2; K refers to the number of parameters in each model.

| Trait | Model number | K | LogLik | AICc | ΔAICc | Weight |
| --- | --- | --- | --- | --- | --- | --- |
| SMR | **5** | **8** | **123.37** | **-227.77** | **0.000** | **0.982** |
|  | 4 | 7 | 118.23 | -219.69 | 8.084 | 0.017 |
|  | 2 | 10 | 117.57 | -211.71 | 16.064 | 0.000 |
|  | 6 | 8 | 111.64 | -204.31 | 23.467 | 0.000 |
|  | 1 | 6 | 107.52 | -200.29 | 27.489 | 0.000 |
|  | 3 | 7 | 105.52 | -194.26 | 33.510 | 0.000 |
|  | 7 | 5 | 11.48 | -12.64 | 215.137 | 0.000 |
| RMR | **5** | **8** | **164.72** | **-310.48** | **0.000** | **0.993** |
|  | 4 | 7 | 158.63 | -300.51 | 9.978 | 0.007 |
|  | 2 | 10 | 156.49 | -294.02 | 16.463 | 0.000 |
|  | 6 | 8 | 158.67 | -293.92 | 15.568 | 0.000 |
|  | 1 | 6 | 151.64 | -288.64 | 21.846 | 0.000 |
|  | 3 | 7 | 149.44 | -282.12 | 28.359 | 0.000 |
|  | 7 | 5 | 44.17 | -78.04 | 232.447 | 0.000 |
| MMR | **4** | **7** | **99.44** | **-182.12** | **0.000** | **0.496** |
|  | **1** | **6** | **98.02** | **-181.45** | **0.670** | **0.355** |
|  | **5** | **8** | **99.07** | **-179.19** | **2.936** | **0.114** |
|  | 3 | 7 | 96.33 | -175.90 | 6.224 | 0.022 |
|  | 6 | 8 | 96.85 | -174.74 | 7.380 | 0.012 |
|  | 2 | 10 | 95.05 | -166.67 | 15.449 | 0.000 |
|  | 7 | 5 | 62.74 | -115.17 | 66.952 | 0.000 |
| AS | **7** | **5** | **25.75** | **-41.19** | **0.000** | **0.783** |
|  | **1** | **6** | **26.27** | **-38.35** | **2.843** | **0.189** |
|  | 3 | 7 | 25.12 | -33.48 | 7.713 | 0.017 |
|  | 4 | 7 | 24.33 | -31.88 | 9.308 | 0.007 |
|  | 6 | 8 | 24.45 | -29.93 | 11.262 | 0.003 |
|  | 5 | 8 | 23.12 | -27.26 | 13.927 | 0.001 |
|  | 2 | 10 | 21.29 | -19.14 | 22.052 | 0.000 |

**Table S3.** Model selection using conditional Akaike information criterion (AICc) values for each behavioural trait. Models for each trait are ranked by cAIC values. Best-fit (ΔAICc < 4) models are bolded. Model parameters are described in Table 2; K refers to the number of parameters in each model.

| Trait | Model number | K | LogLik | AICc | ΔAICc | Weight |
| --- | --- | --- | --- | --- | --- | --- |
| Boldness | **1** | **6** | **-125.23** | **260.76** | **0.000** | **0.455** |
|  | **3** | **6** | **-125.11** | **262.65** | **1.890** | **0.177** |
|  | **4** | **6** | **-125.22** | **262.89** | **2.126** | **0.157** |
|  | **6** | **7** | **-124.39** | **263.36** | **2.600** | **0.124** |
|  | 5 | 7 | -125.11 | 264.80 | 4.039 | 0.069 |
|  | 7 | 3 | -124.38 | 265.51 | 3.755 | 0.049 |
|  | 2 | 9 | -123.76 | 266.48 | 5.712 | 0.026 |
| Activity | **2** | **9** | **-1130.84** | **2282.41** | **0.000** | **0.558** |
|  | **5** | **7** | **-1133.92** | **2283.49** | **1.088** | **0.324** |
|  | **4** | **6** | **-1136.14** | **2285.50** | **3.090** | **0.119** |
|  | 6 | 7 | -1176.96 | 2369.57 | 87.166 | 0.000 |
|  | 3 | 6 | -1178.20 | 2369.62 | 87.215 | 0.000 |
|  | 1 | 5 | -1180.15 | 2371.16 | 88.750 | 0.000 |
|  | 7 | 3 | -1669.84 | 3346.00 | 1063.599 | 0.000 |

**Table S4.** Summary of linear mixed-effects models (LMER) with site ID as a random effect, describing the relationship between log_10_ standard metabolic rate (SMR), log_10_ maximum metabolic rate (MMR), log_10_ routine metabolic rate (RMR), and aerobic scope (AS) and fish log_10_ mass, thermal history, acclimation temperature. Variables and interactions were chosen based on model selection (AICc). Significance is noted as: <0.0001 ‘***’, <0.001 ‘**’, < 0.05 ‘*’.

|  | Estimate | Std. Error | t | df | p |
| --- | --- | --- | --- | --- | --- |
| SMR |  |  |  |  |  |
| Intercept | -2.380 | 0.216 | -11.034 | 187 | <0.0001*** |
| Mass | 0.916 | 0.124 | 7.378 | 184 | <0.0001*** |
| Thermal history | 0.455 | 0.087 | 5.212 | 184 | <0.0001*** |
| Acclimation temperature | 0.0812 | 0.008 | 9.888 | 183 | <0.0001*** |
| Thermal history × Acclimation temperature | -0.018 | 0.003 | -5.635 | 182 | <0.0001*** |
| Mass × Acclimation temperature | -0.023 | 0.005 | -4.487 | 182 | <0.0001*** |
| RMR |  |  |  |  |  |
| Intercept | -1.963 | 0.175 | -11.224 | 190 | <0.0001*** |
| Mass | 0.896 | 0.1009 | 8.876 | 187 | <0.0001*** |
| Thermal history | 0.308 | 0.071 | 4.361 | 167 | <0.0001*** |
| Acclimation temperature | 0.069 | 0.007 | 10.327 | 186 | <0.0001*** |
| Thermal history × Acclimation temperature | -0.127 | 0.003 | -4.826 | 185 | <0.0001*** |
| Mass × Acclimation temperature | -0.019 | 0.004 | -4.777 | 186 | <0.0001*** |
| MMR |  |  |  |  |  |
| Intercept | -1.156 | 0.247 | -4.675 | 188 | <0.0001*** |
| Mass | 0.716 | 0.143 | 4.998 | 188 | <0.0001*** |
| Thermal history | 0.302 | 0.097 | 3.078 | 188 | 0.002** |
| Acclimation temperature | 0.053 | 0.009 | 5.598 | 188 | <0.0001** |
| Thermal history × Acclimation temperature | -0.012 | 0.004 | -3.321 | 186 | 0.0011** |
| Mass × Acclimation temperature | -0.016 | 0.006 | 2.830 | 188 | 0.005** |
| AS |  |  |  |  |  |
| Intercept | -0.044 | 0.154 | 0.288 | 179 | 0.774 |
| Mass | 0.277 | 0.044 | 6.320 | 181 | <0.0001*** |
| Thermal history | -0.004 | 0.032 | 0.123 | 17 | 0.902 |
| Acclimation temperature | 0.012 | 0.003 | 4.437 | 190 | <0.0001*** |

**Table S5.** Summary of *Gambusia affinis* behaviour data as a mixed effect binomial logistic model to model boldness data and a generalised linear mixed-effects model (GLMER) for activity data. Significance is noted as: <0.0001 ‘***’, <0.001 ‘**’, < 0.05 ‘*’.

|  | Estimate | Std. Error | Z | P |
| --- | --- | --- | --- | --- |
| Boldness (*n* = 198) |  |  |  |  |
| Intercept | -0.781 | 1.134 | -0.689 | 0.491 |
| Mass | -0.972 | 0.483 | -2.010 | 0.045* |
| Thermal history | 0.718 | 0.357 | 2.010 | 0.044* |
| Acclimation temperature | 0.061 | 0.031 | 1.995 | 0.046* |
| Activity (*n* = 76) |  |  |  |  |
| Intercept | 5.324 | 0.602 | 8.790 | <0.0001*** |
| Mass | -0.606 | 0.377 | 1.598 | 0.110 |
| Thermal history | -2.011 | 0.808 | 2.473 | 0.013* |
| Acclimation temperature | 0.014 | 0.022 | 0.649 | 0.516 |
| Thermal history ×Mass | 0.650 | 0.500 | 1.292 | 0.196 |
| Acclimation temperature ×Mass | 0.023 | 0.010 | 2.264 | 0.024* |
| Thermal history ×Acclimation temperature | 0.065 | 0.029 | 2.272 | 0.023* |
| Thermal history ×Acclimation temperature ×Mass | -0.031 | 0.013 | 2.321 | 0.020* |

**Table S6.** Analysis of variance (ANOVA) tables, including the factor sex, summarising linear mixed-effects models (LMER) with site ID as a random effect. Metabolic traits are standard metabolic rate (SMR), maximum metabolic rate (MMR), routine metabolic rate (RMR), and aerobic scope (AS). Significance is noted as: <0.0001 ‘***’, <0.001 ‘**’, < 0.05 ‘*’. These models are not the final models used in the text, as sex was excluded following model selection (see Table S4).

|  | Sum Sq | Mean Sq | df | F | P |
| --- | --- | --- | --- | --- | --- |
| SMR |  |  |  |  |  |
| Mass | 1.903 | 1.903 | 1, 191 | 110.693 | <0.0001*** |
| Thermal history | 0.001 | 0.001 | 1, 6 | 0.064 | 0.808 |
| Acclimation temperature | 6.140 | 6.140 | 1, 185 | 357.115 | <0.0001*** |
| Sex | 0.049 | 0.049 | 1, 189 | 2.818 | 0.095 |
| MMR |  |  |  |  |  |
| Mass | 1.395 | 1.395 | 1, 187 | 73.263 | <0.0001*** |
| Thermal history | 0.005 | 0.005 | 1, 6 | 0.288 | 0.610 |
| Acclimation temperature | 2.037 | 2.037 | 1, 188 | 107.029 | <0.001*** |
| Sex | 0.0134 | 0.0134 | 1, 193 | 0.710 | 0.401 |
| RMR |  |  |  |  |  |
| Mass | 2.386 | 2.386 | 1, 193 | 223.176 | <0.0001*** |
| Thermal history | 0.003 | 0.003 | 1, 6 | 0.243 | 0.639 |
| Acclimation temperature | 4.735 | 4.735 | 1, 187 | 442.979 | <0.0001** |
| Sex | 0.052 | 0.052 | 1, 192 | 4.871 | 0.029* |
| AS |  |  |  |  |  |
| Mass | 1.268 | 1.268 | 1, 179 | 31.626 | <0.0001*** |
| Thermal history | 0.001 | 0.001 | 1, 6 | 0.029 | 0.871 |
| Acclimation temperature | 0.667 | 0.667 | 1, 187 | 16.641 | <0.0001*** |
| Sex | 0.000 | 0.000 | 1, 191 | 0.003 | 0.959 |

**Table S7.** Ordinary least squares regression functions (y = a + *b*x) of metabolic rates as a function of body mass for standard metabolic rates (SMR), routine metabolic rates (RMR), and maximum metabolic rates (MMR). We separated models by thermal history and acclimation temperature.

| Measurement | Acclimation temperature | Thermal history | *b* | a | R^2^ | P |
| --- | --- | --- | --- | --- | --- | --- |
| SMR | 20 | Warm | 0.516 | -0.717 | 0.738 | <0.0001 |
|  |  | Ambient | 0.532 | -0.823 | 0.759 | <0.0001 |
|  | 30 | Warm | 0.277 | -0.048 | 0.276 | <0.0001 |
|  |  | Ambient | 0.279 | 0.054 | 0.481 | <0.0001 |
| RMR | 20 | Warm | 0.524 | -0.529 | 0.817 | <0.001 |
|  |  | Ambient | 0.556 | -0.634 | 0.845 | <0.001 |
|  | 30 | Warm | 0.351 | 0.010 | 0.497 | <0.001 |
|  |  | Ambient | 0.329 | 0.133 | 0.638 | <0.001 |
| MMR | 20 | Warm | 0.367 | 0.022 | 0.560 | <0.001 |
|  |  | Ambient | 0.430 | -0.138 | 0.556 | <0.001 |
|  | 30 | Warm | 0.298 | 0.276 | 0.260 | <0.001 |
|  |  | Ambient | 0.178 | 0.554 | 0.350 | <0.001 |

**Table S8.** Summary of censored regression models describing the relationship between emergence latency, mass, and metabolic rate. Metabolic rates are standard metabolic rate (SMR), routine metabolic rate (RMR), and maximum metabolic rate (MMR). Significance is noted as: <0.0001 ‘***’, <0.001 ‘**’, < 0.05 ‘*’.

| **Model** | **Estimate** | **Std Error** | **t** | **P** |
| --- | --- | --- | --- | --- |
| 20°C |  |  |  |  |
| Intercept | -400.25 | 498.36 | -0.803 | 0.422 |
| SMR | -832.35 | 542.58 | -1.534 | 0.125 |
| Mass | 768.31 | 335.21 | 2.292 | 0.022* |
| Intercept | -177.50 | 496.58 | -0.357 | 0.721 |
| RMR | -719.82 | 692.27 | -1.040 | 0.298 |
| Mass | 724.36 | 417.87 | 1.733 | 0.083. |
| Intercept | 241.14 | 298.98 | 0.807 | 0.420 |
| MMR | 80.27 | 440.20 | 0.182 | 0.855 |
| Mass | 307.02 | 250.45 | 1.266 | 0.220 |
| 30°C |  |  |  |  |
| Intercept | 481.89 | 207.87 | 2.318 | 0.020* |
| SMR | 548.39 | 300.06 | 1.828 | 0.068. |
| Mass | -81.03 | 147.62 | -0.549 | 0.583 |
| Intercept | -874.89 | 778.58 | -1.124 | 0.2611 |
| RMR | 2752.03 | 1226.66 | 2.244 | 0.025* |
| Mass | 605.27 | 477.66 | 1.267 | 0.205 |
| RMR ×Mass | -1159.70 | 659.74 | -1.758 | 0.079. |
| Intercept | 297.29 | 237.21 | 1.253 | 0.210 |
| MMR | 491.22 | 291.83 | 1.683 | 0.092. |
| Mass | -53.58 | 143.70 | -0.373 | 0.709 |

**Table S9.** Summary of a Poisson-lognormal generalised linear mixed-effects models describing the relationship between activity, mass, and metabolic rate. Metabolic rates are standard metabolic rate (SMR), routine metabolic rate (RMR), and maximum metabolic rate (MMR). Significance is noted as: <0.0001 ‘***’, <0.001 ‘**’, < 0.05 ‘*’.

| Model | Estimate | Std Error | t | p |
| --- | --- | --- | --- | --- |
| 20°C |  |  |  |  |
| Intercept | 5.50 | 0.30 | 18.333 | <0.0001*** |
| SMR | 0.21 | 0.27 | 0.773 | 0.439 |
| Mass | -0.51 | 0.12 | -4.130 | <0.0001*** |
| Intercept | 3.55 | 0.38 | 9.447 | <0.0001*** |
| RMR | 0.01 | 0.66 | 0.017 | 0.986 |
| Mass | 1.15 | 0.23 | 5.106 | <0.0001*** |
| RMR × Mass | -1.34 | 0.39 | -3.431 | 0.0006*** |
| Intercept | 3.69 | 0.51 | 7.291 | <0.0001*** |
| MMR | 1.69 | 0.68 | 2.490 | 0.013* |
| Mass | 1.10 | 0.31 | 3.542 | 0.0004*** |
| MMR × Mass | -1.82 | 0.45 | -4.007 | <0.0001*** |
| 30°C |  |  |  |  |
| Intercept | 5.73 | 0.07 | 83.242 | <0.0001*** |
| SMR | 0.020 | 0.09 | 0.227 | 0.820 |
| Mass | -0.13 | 0.04 | -3.208 | 0.001** |
| Intercept | 5.72 | 0.07 | 85.181 | <0.0001*** |
| RMR | -0.13 | 0.14 | -0.922 | 0.357 |
| Mass | -0.08 | 0.06 | -1.237 | 0.216 |
| Intercept | 5.74 | 0.07 | 79.106 | <0.0001*** |
| MMR | -0.04 | 0.08 | -0.484 | 0.629 |
| Mass | -0.12 | 0.04 | -2.994 | 0.003** |


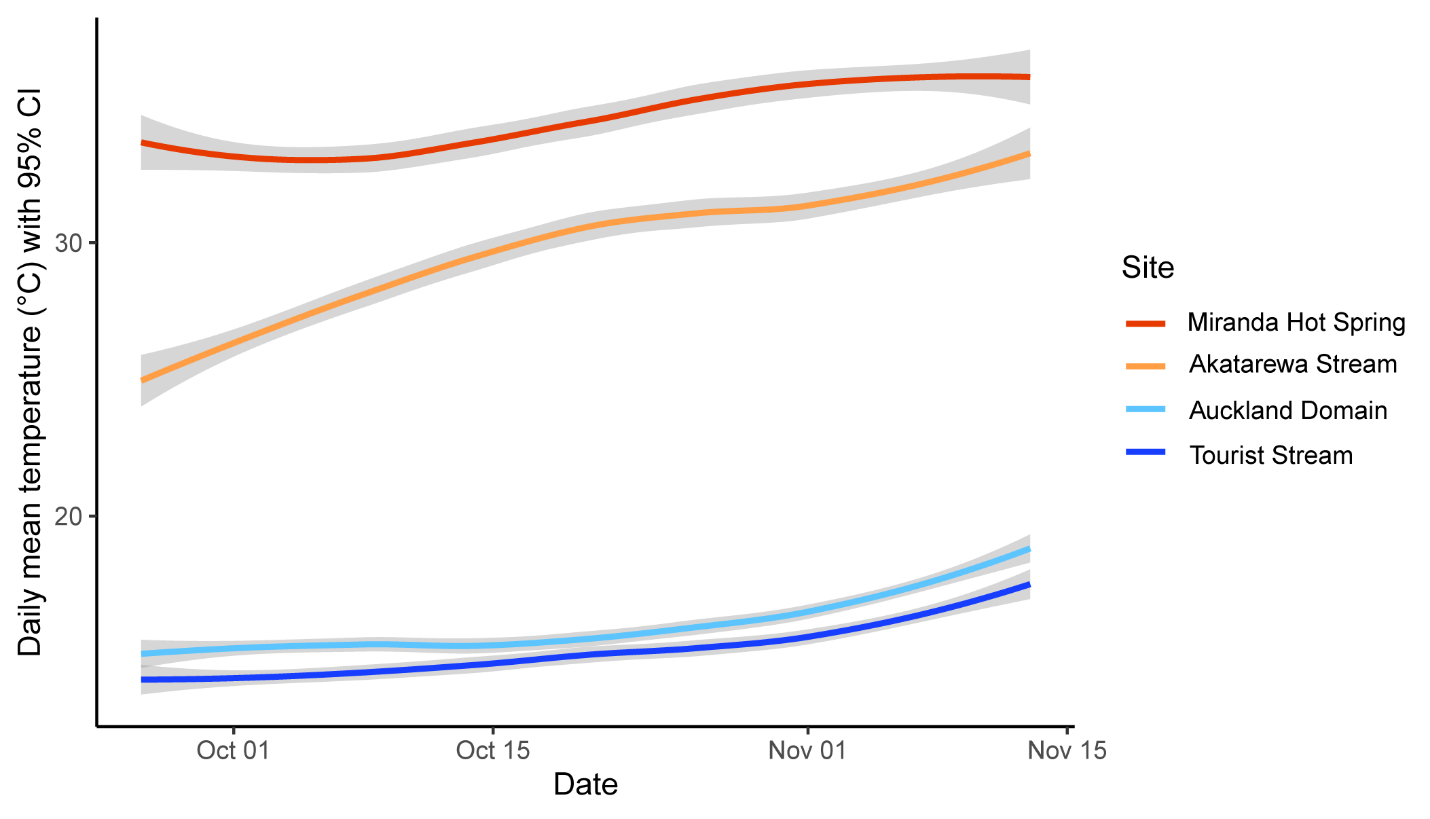


**Figure S1.** Temperature logger data from two geothermal (Akatarewa Stream, Miranda Hot

Spring) and two ambient (Auckland Domain, Tourist Stream) locations used in this study.


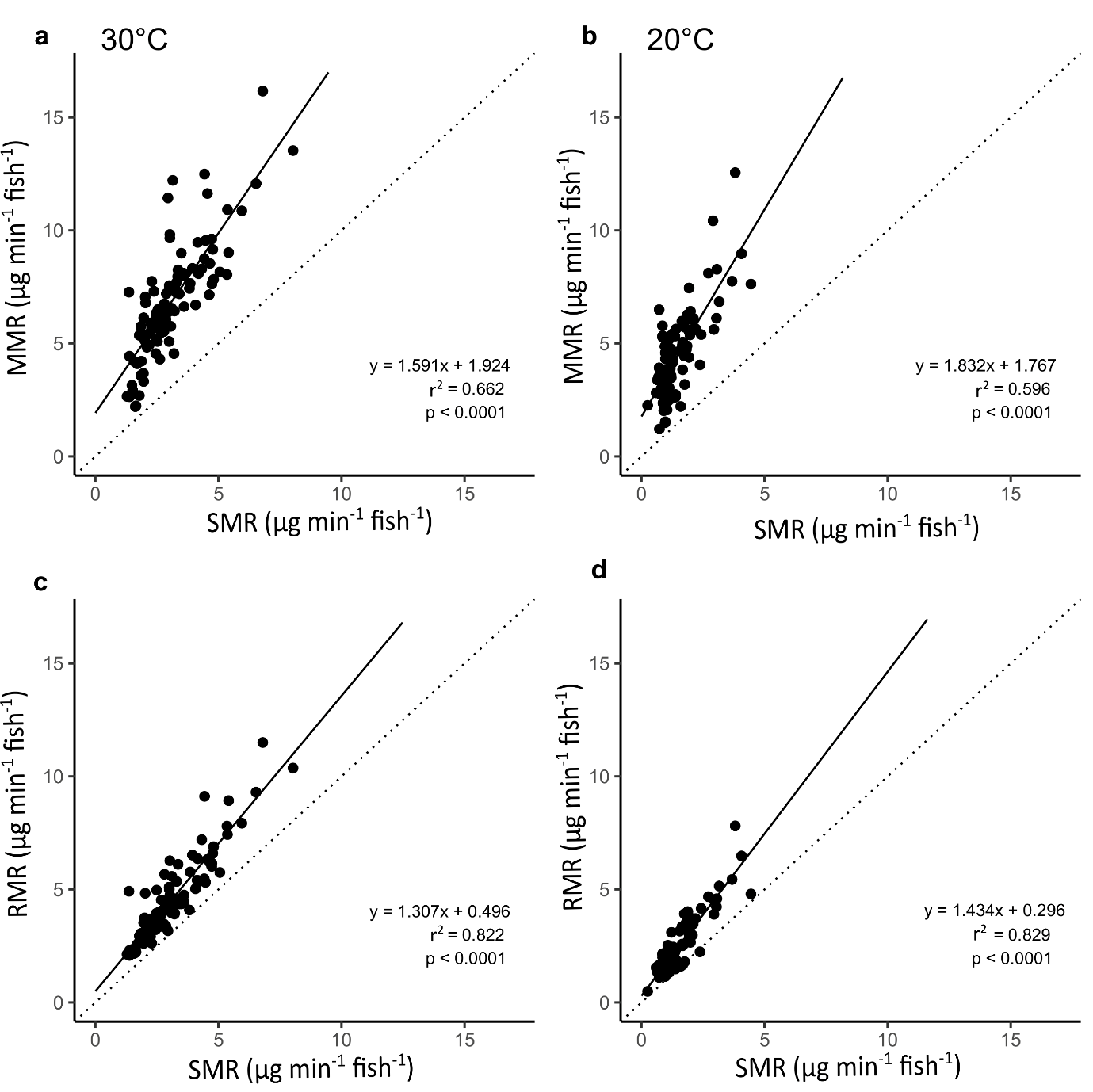


**Figure S2.** Comparison of metabolic rate data measured as MMR, RMR, and SMR. We separated data by laboratory acclimation temperature. Data points represent individual fish. *N* = 198.


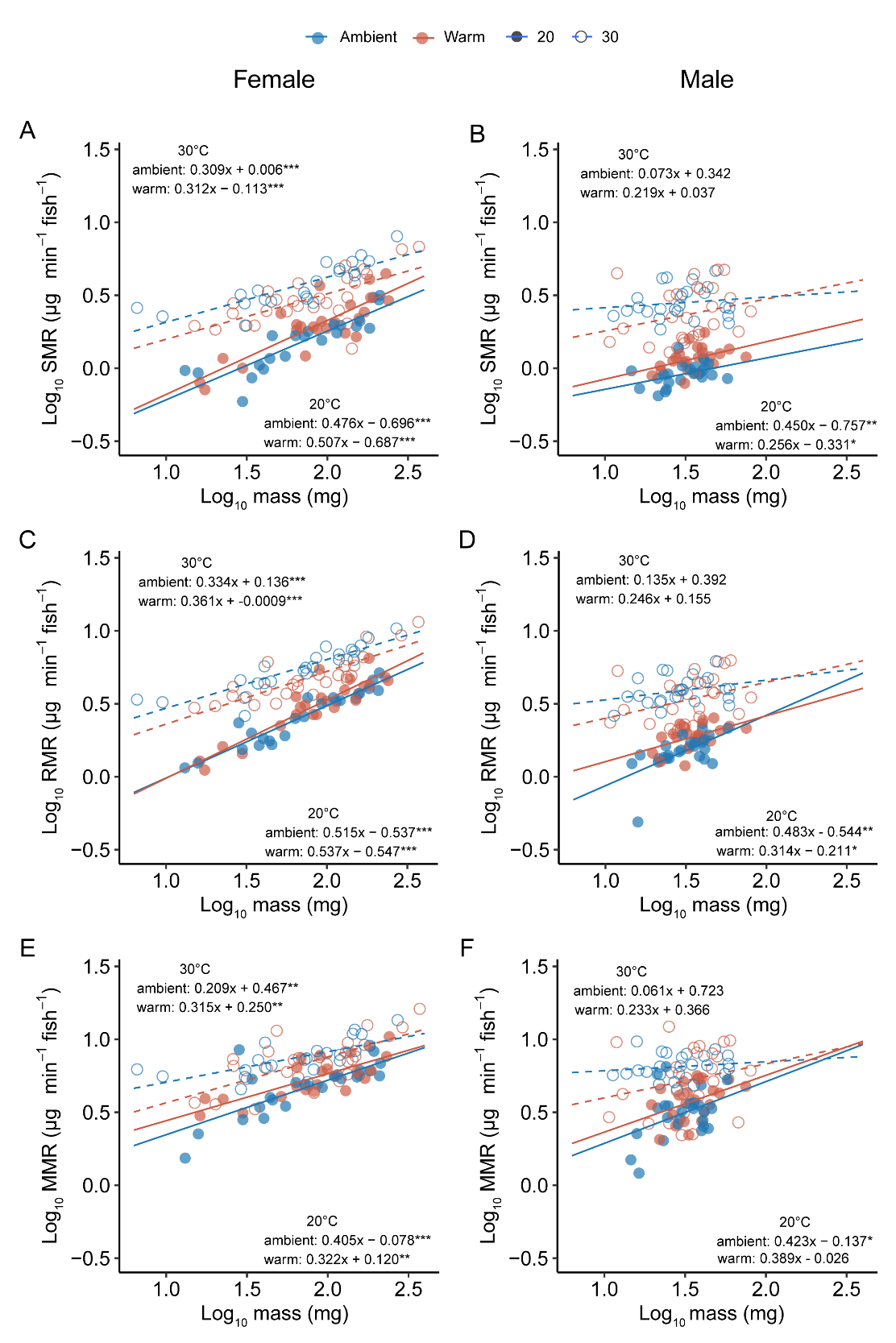


**Figure S3.** Relationship between fish mass and A, B) standard metabolic rate (SMR) C, D) routine metabolic rate (RMR), and E, F) maximum metabolic rate (MMR) in *Gambusia affinis* from ambient and warm populations, where data are split by sex.


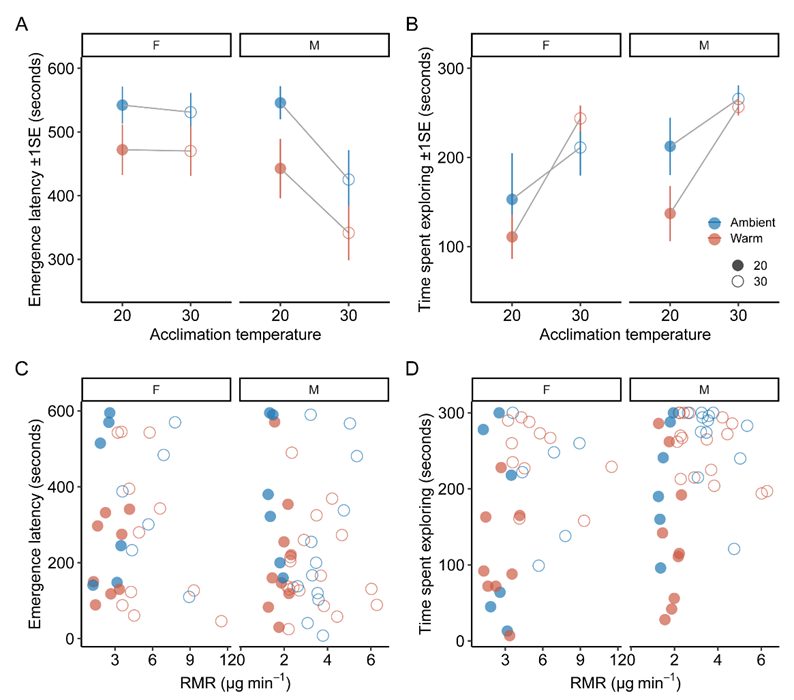


**Figure S4**. Relationship between thermal history and behaviour as A) boldness (i.e., emergence latency) and B) activity (i.e., time spent exploring) as population differences. Plots C and D show individual differences across all populations between routine metabolic rate (RMR), and behaviour as boldness and activity, respectively. We do not show individuals who did not leave the refuge, *n* = 76. All data are split by sex.
